# Presynaptic Mechanisms of Lead Neurotoxicity: Effects on Vesicular Release, Vesicle Clustering and Mitochondria Number

**DOI:** 10.1371/journal.pone.0127461

**Published:** 2015-05-26

**Authors:** Xiao-lei Zhang, Sara R. Guariglia, Jennifer L. McGlothan, Kirstie H. Stansfield, Patric K. Stanton, Tomás R. Guilarte

**Affiliations:** 1 Department of Cell Biology & Anatomy, New York Medical College, Valhalla, New York, United States of America; 2 Department of Environmental Health Sciences, Mailman School of Public Health, Columbia University, New York, New York, United States of America; Institute for Health & the Environment, UNITED STATES

## Abstract

Childhood lead (Pb^2+^) intoxication is a global public health problem and accounts for 0.6% of the global burden of disease associated with intellectual disabilities. Despite the recognition that childhood Pb^2+^ intoxication contributes significantly to intellectual disabilities, there is a fundamental lack of knowledge on presynaptic mechanisms by which Pb^2+^ disrupts synaptic function. In this study, using a well-characterized rodent model of developmental Pb^2+^ neurotoxicity, we show that Pb^2+^ exposure markedly inhibits presynaptic vesicular release in hippocampal Schaffer collateral-CA1 synapses in young adult rats. This effect was associated with ultrastructural changes which revealed a reduction in vesicle number in the readily releasable/docked vesicle pool, disperse vesicle clusters in the resting pool, and a reduced number of presynaptic terminals with multiple mitochondria with no change in presynaptic calcium influx. These studies provide fundamental knowledge on mechanisms by which Pb^2+^ produces profound inhibition of presynaptic vesicular release that contribute to deficits in synaptic plasticity and intellectual development.

## Introduction

Childhood lead (Pb^2+^) intoxication continues to be a public health problem of significant proportion not only in the United States, but also globally [1,2]. Many studies over several decades have consistently demonstrated that one of the most prominent effects of Pb^2+^ in children is to decrease their capacity to learn with devastating effects on cognitive and intellectual development [3,4,5,6,7]. The consequences of childhood Pb^2+^ intoxication on the intellectual capacity of children and society as a whole are immeasurable in a world dominated by an economy that rewards knowledge. Recent human studies have also shown that Pb^2+^ exposure early in life is associated with longitudinal declines in cognitive function [8] and loss of brain volume [9,10] in aging individuals. Therefore, Pb^2+^ exposure in early life has immediate and long-term consequences to neurological and mental health.

Neuronal chemical communication is mediated by synapses that undergo fast and efficient release of neurotransmitters. Neurotransmitter release occurs at presynaptic active zones (PAZ) which are specialized regions of the synapse juxtaposed to postsynaptic densities (PSD). In presynaptic terminals, neurotransmitters are packaged in synaptic vesicles that are organized in clusters or functional pools that include: 1) the readily-releasable/docked vesicle pool, 2) the rapidly recycling pool from which vesicles undergo exo-endocytosis as a result of stimulation and, 3) the resting pool, which serves as a reservoir containing vesicles that are released-reluctant unless there is sustained and strong stimulation [11,12]. Disruption of synaptic function is known to result in neurological disease [13,14].

A number of studies have shown that acute and chronic exposure to Pb^2+^ alters neurotransmitter release in both *in vivo* models and *in vitro* systems. Pb^2+^ decreases evoked release of glutamate (Glu) and gamma-aminobutyric acid (GABA) in young adult rats developmentally exposed to Pb^2+^ [15] and in hippocampal cultures and brain slices acutely exposed to Pb^2+^ [16, 17]. Both spontaneous and action potential-evoked release of Glu and GABA are affected by Pb^2+^ exposure [18], but there is a paucity of knowledge of the mechanisms underlying these effects. Recent studies from our laboratory have provided the first working model by which exposure to Pb^2+^ during the period of synaptogenesis affects synapse development and function that can explain effects of Pb^2+^ on both presynaptic and postsynaptic compartments of the synapse [19,20,21]. Using a Pb^2+^ exposure paradigm during the period of synaptogenesis in primary hippocampal neuron cultures, we found that Pb^2+^ inhibition of postsynaptic NMDA receptors (NMDAR) alters downstream calcium signaling and impairs the CREB-dependent transcription of activity-regulated genes, such as brain-derived neurotrophic factor (BDNF) and alters the function of its cognate receptor TrkB and downstream signaling to modify synapsin I phosphorylation at serine sites involved in vesicle movement [19,20,21]. These studies also showed that the Pb^2+^-induced impairment of BDNF trans-synaptic retrograde signaling decreased the levels of the vesicular proteins synaptophysin and synaptobrevin and inhibited vesicular release [19]. Importantly, the addition of exogenous BDNF normalized synaptophysin and synaptobrevin protein levels and reversed the impairment in vesicular release providing the first evidence of the beneficial effects of BDNF on Pb^2+^-induced synaptic dysfunction [19]. We also showed that the inhibition of vesicular release by Pb^2+^ was specific to a fast-releasing pool of vesicles that we hypothesized to be the rapidly-releasable/docked vesicle pool [19].

To determine whether the effects of Pb^2+^ exposure that we have observed using *in vitro* exposure of hippocampal neurons in culture is operational in the hippocampus of young adult rats that have been chronically exposed to Pb^2+^ *in vivo*, we performed electrophysiological and two-photon imaging studies in Schaffer collateral-CA1 synapses. Further, to identify the subcellular basis of the Pb^2+^-induced impairment of vesicular release we used transmission electron microscopy (TEM) to measure vesicle number in the different vesicle pools of the presynaptic compartment as well as presynaptic mitochondria number and size. We report here that chronic *in vivo* exposure to Pb^2+^ during development resulted in a marked inhibition of Schaffer-collateral-CA1 synaptic transmission by inhibiting vesicular release of glutamate, an effect that was not associated with a persistent change in presynaptic calcium entry. On the other hand, we found a reduced number of synaptic vesicles associated with the rapidly- releasable/docked vesicle pool confirming our hypothesis originating from previous *in vitro* studies [19] that the inhibitory effect of Pb^2+^ on vesicular release was due to, at least in part, reductions in the number of vesicles in the rapidly releasable/docked vesicle pool. Furthermore, we observed an increase in the dispersion of vesicles (nearest neighbor distance) in the resting pool and a decrease in the number of presynaptic terminals containing multiple mitochondria. The latter suggests that energy availability in the form of ATP may also be compromised by Pb^2+^ exposure and influence vesicular release.

## Materials and Methods

### Chemicals

Salts were acquired from Sigma-Aldrich, CNQX from Tocris, and FM1-43 from Invitrogen. All electron microscopy supplies (EM grade paraformaldehyde (10% aq), EM grade glutaraldehyde (10% aq), uranyl acetate (4% aq), osmium tetroxide (4% aq), ethanol, propylene oxide, Spurr Low Viscosity Resin Kit, 200 mesh copper grids, lead acetate) were acquired from Electron Microscopy Sciences (EMS Diasum, Hatfield, PA).

#### Blood Pb^2+^ analysis.

Blood Pb^2+^ analysis was conducted using the LeadCare system (ESA Laboratories, Inc, Chelmford, MA) as described by the manufacturer.

### Animals

Adult female Long-Evans rats were purchased from Charles River, Inc. (Wilmington, MA) and fed 0 (control) or 1500 ppm lead acetate (PbAc) in the diet (Dyets, Bethlehem, PA) 10 days prior to breeding with non-exposed, normal Long-Evans males. Litters were culled to 10 on postnatal day 1 (PN1) and dams were maintained on their respective diet until weaning of pups. After weaning, offspring remained on their respective maternal diet until PN50. At weaning, rats were housed in same sex pairs in plastic cages at 22 ± 2°C on a 12/12 light: dark cycle. Food and water were allowed ad libitum. Litters of rats were considered a single experimental unit for statistical purposes so that for each experiment, one animal was used from a single litter for one data point. This study was carried out in strict accordance with the recommendations in the Guide for the Care and Use of Laboratory Animals of the National Institutes of Health. The protocol was approved by Columbia University and New York Medical College Institutional Animal Care and Use Committees (AC-AAAF4810). All non-survival procedures were performed under sodium pentobarbital anesthesia, and all efforts were made to minimize suffering.

### Hippocampal slice electrophysiology

Experiments were conducted as described previously [22,23]. At 50 ± 2 days of age, rats were deeply anesthetized with isoflurane, decapitated and their brains rapidly removed and submerged in ice-cold artificial cerebrospinal fluid (ACSF, 2–4°C), containing (in mM): 124 NaCl, 4 KCl, 2 MgSO_4_, 2 CaCl_2_, 1.25 NaH_2_PO_4_, 26 NaHCO3, 10 glucose; at pH 7.4, gassed continuously with 95% O_2_/5% CO_2_). Brains were hemisected, the frontal lobes cut off, and individual hemispheres glued using cyanoacrylate adhesive onto a stage immersed in ice-cold ACSF gassed continuously with 95% O_2_/5% CO_2_ during slicing. We cut 400 μm thick coronal slices using a vibratome (Leica VT1200S), and transferred them to an interface holding chamber for incubation at room temperature for a minimum of 1 hr before transferring to a submerged recording chamber continuously on a Zeiss Axioskop microscope continuously perfused at 3 ml/min with oxygenated ACSF at 32 ± 0.5°C.

Whole cell patch-clamp recordings were performed in CA1 pyramidal neurons using standard techniques. Patch pipettes (R = 3–4 MΩ) were filled with recording solution containing (mM): 135 CsMeSO_2_, 8 NaCl, 10 HEPES, 2 Mg-ATP, 0.3 Na-GTP, 0.5 EGTA, and 1 QX-314 (275 mOsm, pH = 7.25 adjusted with Cs(OH)_2_). Access resistance was carefully monitored, and only cells with stable access resistance (<5% change) were included in analyses. CA1 pyramidal cells were recorded under voltage clamp using a MultiClamp 700B (Axon Instruments) with Clampex (v9). Recording signals were filtered through an eight-pole Bessel low-pass filter with a 3 kHz cutoff frequency, digitized at 10 kHz, and sampled using Clampex (v9). Sampled data was analyzed off line with Clampfit (v9) and OriginPro (v6.1). Neurons were clamped at –60mV, and Schaffer collateral-evoked EPSCs were triggered by MultiClamp and delivered by a bipolar stimulating electrode (FHC, USA) via a stimulus isolator (ISO-Flex, AMPI Instruments; 50–100 pA, 100 μs duration). EPSC slopes were calculated by linear interpolation of the initial downward current from 20% to 80% of the maximum EPSC amplitude. Paired-pulse facilitation was assessed by applying a pair of Schaffer collateral stimuli at intervals of 10–125 msec, and the ratio of slopes of the second to the first response was calculated, so that numbers greater than 1.0 represented facilitation, less than 1.0 inhibition. Chemicals for making extra- and intracellular solutions were purchased from Sigma (USA) or Fluka (USA).

### Two-photon laser scanning microscopy

Fluorescence was visualized using a customized two-photon laser-scanning Olympus BX61WI microscope with a 60x/0.90W water immersion infrared objective lens and an Olympus multispectral confocal laser scan unit. The light source was a Mai-Tai™ laser (Solid-State Laser Co., Mountain View, CA), tuned to 820 nm for exciting Magnesium Green and FM1-43. Epifluorescence was detected with photomultiplier tubes of the confocal laser scan head with pinhole maximally opened and emission spectral window optimized for signal over background. In the transfluorescent pathway, a 565 nm dichroic mirror was used to separate green and red fluorescence to eliminate transmitted or reflected excitation light (Chroma Technology, Rockingham, VT). Depending on the nature of the fluorescent dyes, HQ525/50 and HQ610/50 or HQ710/50 filters were placed in the “green” and “red” pathways, respectively. Image acquisition was controlled by Fluoview FV300 software (Olympus America, Melville, NY). After confirming the presence of Schaffer collateral-evoked fEPSPs >1mV in amplitude in CA1 *stratum radiatum*, and inducing LTP, 10 μM 6-cyano-7-nitroquinoxaline-2,3-dione (CNQX) was bath-applied throughout the rest of the experiment to prevent synaptically-driven action potentials in CA3 pyramidal neurons from accelerating dye release. Presynaptic boutons were loaded by bath-applying 5μM FM1-43 (Molecular Probes) in hypertonic ACSF supplemented with sucrose to 800mOsm for 25 sec to selectively load the rapidly-recycling pool (RRP) [24,25], then returned to normal ACSF. Stimulus-induced destaining was measured after 30 min perfusion with dye-free ACSF, by bursts of 10Hz bipolar stimuli (150 μs DC pulses) for 2 sec applied once each 30 sec. We fitted a single exponential to the first 6 fluorescence time course values, and taus between groups compared by two-tailed Student’s t-test, as we have shown previously that the early release reflects vesicular release from the RRP prior to recycling and reuse of vesicles [24,25]. At the end of each experiment, complete depolarization-induced destaining was evoked by bath-applying 85 mM [K^+^] ACSF. Using established methods for measuring [Ca^2+^] transients [26], we filled Schaffer collateral presynaptic fibres with Magnesium Green AM. Briefly, an ejection electrode (tip diameter, 5–10 μm) containing Magnesium Green AM (1mM Magnesium Green AM, 10% DMSO, 1% pluronic acid in ACSF) was lowered into the Schaffer collateral pathway between the stimulating electrode and the presynaptic terminal field to be observed, air pressure pulses (6–9 psi, 100–200 msec) controlled by a Picospritzer (General Valve Corp. USA) were applied to the electrode until a small bright spot (≈10 mm in diameter) was observed. Then the slice was maintained with a 3ml/min flow of oxygenated ACSF for ~30 minutes to allow the dye to sufficiently diffuse into presynaptic boutons. To verify that magnesium green selectively loaded into presynaptic terminals, FM4-64 was loaded with high [K^+^]o at the end of each experiment. To measure Ca^2+^ dynamics the fluorescence was collected by scanning at 200 Hz in a surface-scanning mode (XYT). Baseline fluorescence (F_0_) was the average of four images during control, *Δ*F/F was calculated as (*Δ*F/F)_(*t*)_ = (F_(*t*)_-F_0_)/F_0_.

### Transmission electron microscopy specimen preparation

At PN50, rats were anesthetized with 20 mg/kg pentobarbital. Rats were perfused transcardially with 2.5% glutaraldehyde + 2% paraformaldehyde in 0.1 M Phosphate Buffered (PBS). The brain was removed and post-fixed in the same solution overnight at room temperature (RT). Brains were sectioned into 500 μm slices with a vibratome. Tissue from the same CA1 region in which the electrophysiological studies were performed were dissected from the hippocampus using a 1.5 mm hole-punch. Dissected tissue was placed in 2.5% glutaraldehyde + 2% paraformaldehyde in PBS mixture for 3 hr at RT and rinsed with PBS. Secondary fixation in 1% osmium tetroxide in PBS was done for 60 min at RT. Following osmium fixation, tissue was rinsed in PBS then rinsed in water to remove all traces of phosphate from samples. Tissue was subsequently dehydrated in 50% ethanol, a mixture of 70% ethanol + 1% uranyl acetate, 85% ethanol and 2 changes of 100% ethanol (15 min per step). Tissue was then placed in the transition solvent propylene oxide twice (15 min per step) and was left to infiltrate in a 1:1 mixture of propylene oxide-Spurr’s Resin overnight at RT. Steps involving osmium tetroxide and uranyl acetate were done in containers covered with foil to block light. Tissue was transferred to pure Spurr’s Resin for infiltration for 24 hours at RT. Tissue was then placed into Beem Capsules with fresh Spurr’s Resin, allowed to sit for 30 min and then placed in a 70°C oven for 24 hours for polymerization. After polymerization, ultrathin sections (70 nm) were obtained using a Sorval 5000 ultramicrotome and placed onto 200 mesh copper grids. 2 μm of tissue was cut in between each collected section to prevent repeat analysis of any synapses. Sections on grids were stained with uranyl acetate for 45 min, rinsed with water, stained with lead citrate for 90 sec, rinsed again with water and left to dry on clean filter paper.

### Transmission electron microscopy

Tissue was examined under a Hitachi 7500 Transmission Electron Microscope operated at 80 kV. Images were obtained at 100,000x magnification using an AMT digital camera and software. For each hippocampi under investigation (10 total; 5 Control and 5 Pb^2+^), a total of 40 images of simple, asymmetric synapses were obtained. Five synapses were imaged from each grid. Synapses were spaced by a minimum of one grid box to reduce bias. The microscopist was blinded to treatment conditions while imaging.

### Transmission electron microscopy image analysis

The presynaptic active zone (PAZ) and the center of each pre-synaptic vesicle were marked using ImageTool (UTHSCSA, ImageTool, Version 3.0). The distance between each vesicle and the PAZ as well as the distance in between each vesicle and its nearest neighbor was calculated using ImageTool coordinates in LoClust [27]. The diameter of each vesicle was measured using ImageJ. The PAZ length was also measured using ImageJ. PAZ membrane appears more electron dense after staining than surrounding membranes, which allows for measurement. The postsynaptic density (PSD) length was measured using ImageJ. The PSD is large and electron dense after staining, which facilitates measurement. Vesicles were classified as readily-releasable/docked vesicle pool if they were physically contacting the PAZ. Vesicles were classified as belonging to the recycling pool if their center was within 200 nm of the PAZ. Vesicles were considered part of the reserve pool if their vesicular center was greater than 200 nm from the PAZ. The number and diameter of mitochondria in pre-synaptic terminals was also determined. All measurements were made by one individual (SRG) who was blinded to the treatment groups.

### Statistics

Forty EM images were obtained from each Schaffer Collateral—CA1 region. An average of each measurement made from the 40 images was used to represent the animal. A t-test with Welch Correction was used to determine differences between the control and Pb^2+^ treated groups for each particular measure (GraphPad Prism). Welch correction was used to account for differences in variance. In analyses requiring comparisons between multiple groups, an ANOVA with Sidak’s Multiple Comparisons analysis was used, to determine which groups were significantly different than one another. Significance level was preset to *p* < 0.05.”

## Results

### Blood lead levels and body weight of rats

The Pb^2+^ exposure paradigm used in the present study does not produce any overt toxicity based on body weight gain. Body weight at postnatal day 50 (PN50) were: 294.4 ± 4.8 grams (n = 24) for control animals and 281.6 ± 6.9 grams for Pb^2+^-exposed animals.

Blood Pb^2+^ levels of littermates to animals used in this study at PN50 were: 0.8 ± 0.3 μg/dL (n = 11) for control animals and 21.1 ± 1.6 μg/dL (n = 15) for Pb^2+^-exposed animals. This level of Pb^2+^ exposure is environmentally relevant and previous studies using this animal model have shown that it produces in deficits in synaptic plasticity in the form of long-term potentiation [28], decreased adult neurogenesis with alterations in the morphological development of newly born granule cells in the hippocampus [29], and impairments of spatial learning and contextual fear conditioning [28,30,31] in animals of similar age.

### Chronic lead exposure persistently enhances paired-pulse facilitation at Schaffer collateral-CA1 synapses

**Fig 1:**
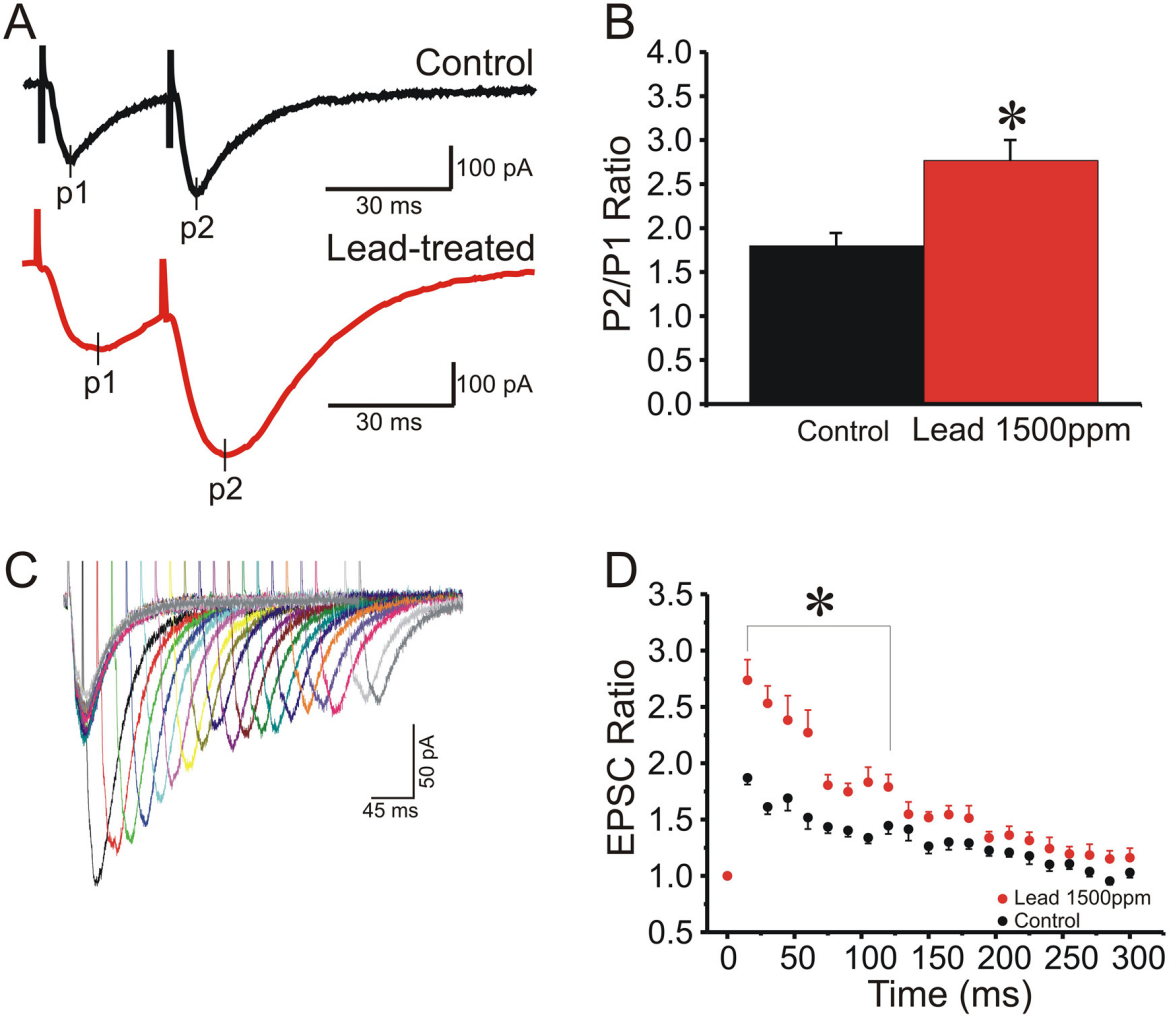
Schaffer collateral-CA1 paired-pulse facilitation (PPF) is enhanced in chronically Pb^2+^-exposed rat hippocampus, indicative of reduce transmitter release. (A) Representative excitatory postsynaptic currents (EPSC) in field CA1 in response to Schaffer collateral paired-pulse stimuli at a 30 ms interstimulus interval (ISI) in a hippocampal slice from a control (black trace) versus a Pb^**2+**^-treated (red trace) rat. (B) Mean ± SEM ratio of P2/P1 (30ms ISI) at Schaffer collateral-CA1 synapses in slices from control (black, N = 13 slices) versus Pb^**2+**^-treated (red, N = 9 slices) rats, showing that PPF was significantly larger in Pb^**2+**^-treated rats (*, *P*<0.05, Student’s t-test). (C) Representative EPSCs evoked by the second of two Schaffer collateral stimuli in a CA1 pyramidal neuron, illustrating the ISI range of PPF. (D) Mean ± SEM EPSC PPF P2/P1 ratio as a function of ISI, where PPF was significantly enhanced for ISI 10–125 ms in slices from Pb^**2+**^-treated (red circles) versus control (black circles) rats (*, *P*<0.05, ANOVA for repeated measures and post-hoc Student’s t-test with Bonferroni correction).

Neuronal short-term presynaptic plasticity is classically assessed with “paired-pulse stimulation,” two stimuli in close succession [32,33]. One form of paired-pulse modulation, paired pulse facilitation (PPF), is typically attributed to an increase of release probability (*Pr*) during the second stimulus, arising from prior accumulation of residual Ca^2+^ near active zones and/or lingering effects of Ca^2+^ on a Ca^2+^ sensor [33,34]. This residual Ca^2+^, when present at terminals that *fail* to release on the first stimulus, will cause them to release and increase response amplitude from the second stimulus. Therefore, if initial *Pr* is reduced, as by manipulations such as reducing extracellular [Ca^2+^], the magnitude of PPF (the ratio of second to first response amplitude) will be increased [33,34]. As Fig 1A illustrates, PPF in CA1 pyramidal neurons was readily elicited by two Schaffer collateral stimuli applied with a 30 ms interpulse interval. As summarized in Fig 1B, the ratio of excitatory postsynaptic current (EPSC) amplitude in response to the second stimulus versus the first stimulus (P2/P1) was significantly greater in slices from Pb^2+^-exposed animals compared to controls, consistent with a decrease in initial *Pr*. When paired-pulse stimuli were applied at intervals that varied from 20–300 msec, the significant increase in PPF in slices from Pb^2+^-exposed rats was statistically significant for interpulse intervals from 20–125 msec (Fig 1C and 1D; *P* <0.05, repeated measures ANOVA with post-hoc Student’s t-test with Bonferroni correction).

### Chronic lead exposure persistently reduces vesicular release from the rapidly-recycling vesicle pool at Schaffer collateral presynaptic terminals

**Fig 2:**
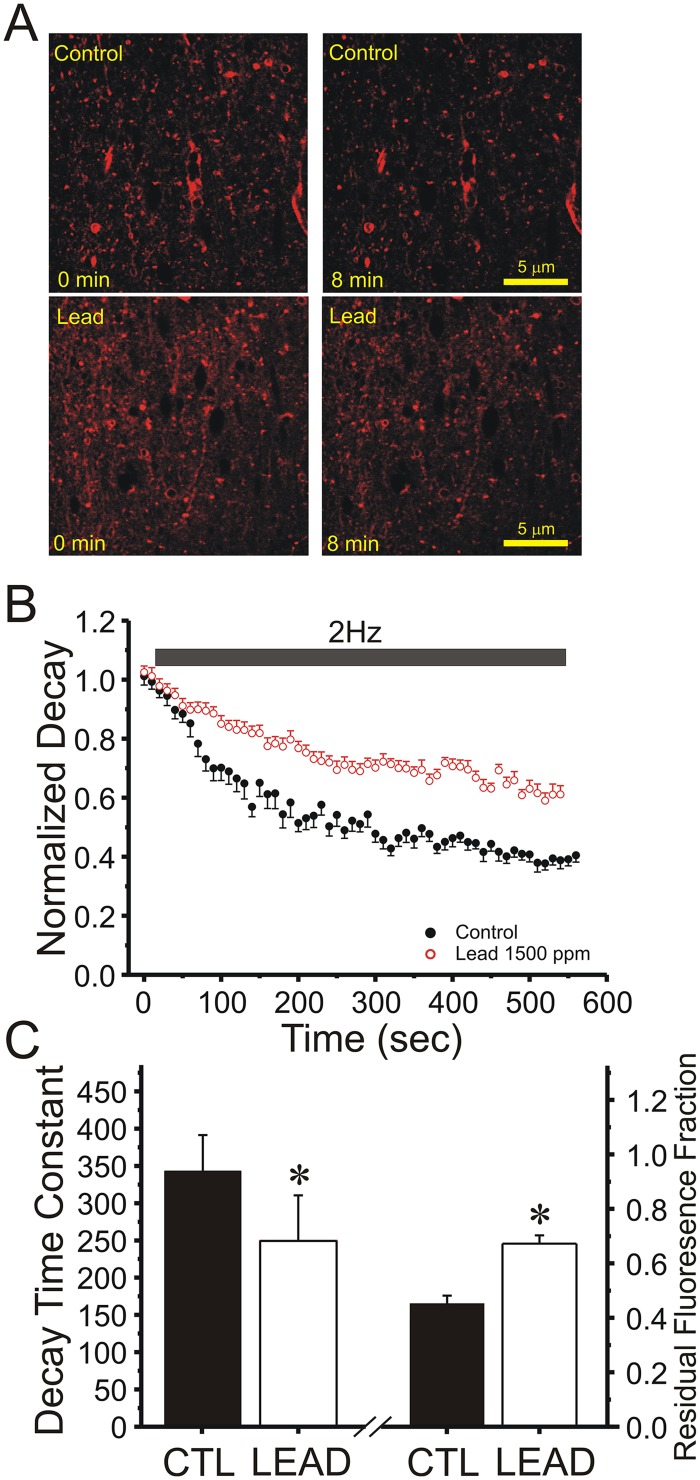
Two-photon laser scanning microscopic (TPLSM) images of FM1-43 vesicular release from Schaffer collateral terminals in field CA1 of hippocampal slices confirm that release probability is reduced by chronic Pb^2+^ exposure. (A) Representative TPLSM pseudocolor images of FM1-43 loaded presynaptic terminals in *stratum radiatum* of field CA1 in hippocampal slices from Pb^**2+**^-treated and control rats imaged prior to (0 min) and after 8 minutes (8 min) of 2Hz Schaffer collateral stimulation (Calibration Bar: 5 μm). (B) Time course (Mean ± SEM) of stimulus-evoked FM1-43 destaining from puncta in field CA1 of hippocampal slices in response to 2Hz Schaffer collateral stimulation (solid bar) in control (black circles, N = 8 slices, 35 puncta) versus Pb^**2+**^-treated (red circles, N = 6 slices, 30 puncta) rats. (C) Mean ± SEM of initial decay time constant (left bars) and ending residual fluorescence (right bars) in the slices in (B) from control (CTL) versus Pb^**2+**^-treated (LEAD) rats (*,*P* <0.05, Student’s t-test), showing slower destaining of Schaffer collateral terminals from rats chronically exposed to Pb^**2+**^.

To determine directly whether presynaptic vesicular release is altered by chronic *in vivo* Pb^2+^ exposure, we used two-photon excitation to visualize release of the styryl dye FM1-43 from the rapidly-recycling pool of presynaptic vesicles after selective loading by hypertonic shock into Schaffer collateral-CA1 terminals in hippocampal slices. In these experiments, presynaptic vesicles in the rapidly-recycling pool are first stimulated by a brief hypertonic shock to fuse with the membrane and release their transmitter, whereupon they take up FM1-43 from the extracellular space and are endocytosed and recycled into the rapidly-recycling pool for the next evoked release. We have used this method previously to show that generation of LTP and LTD can be associated with persistent increases [24] and decreases [25] in the rate of stimulus-evoked FM1-43 destaining at Schaffer collateral terminals in field CA1. Fig 2 shows the effect of chronic Pb^2+^ exposure on vesicular release from Schaffer collateral presynaptic terminals. Fig 2A shows representative pseudocolor images of FM1-43 labelled Schaffer collateral terminals before (0 min) and after 8 minutes of 2Hz stimulation in control versus slices from Pb^2+^-exposed rats. Fig 2B summarizes the time course of all slices, showing the markedly slower vesicular release evoked by 2Hz stimulation of Schaffer collateral terminals in field CA1 of slices from Pb^2+^-exposed rats compared to control rats. The initial time constant of release calculated from a single exponential fit of the first 6 times points [24,25] was significantly slower (Student’s t-test, *P*<0.05), while the residual fluorescence at the end of the stimulus train was significantly higher in slices from chronically Pb^2+^-exposed rats, confirm a slower rate of vesicular release (Fig 2C).

**Fig 3:**
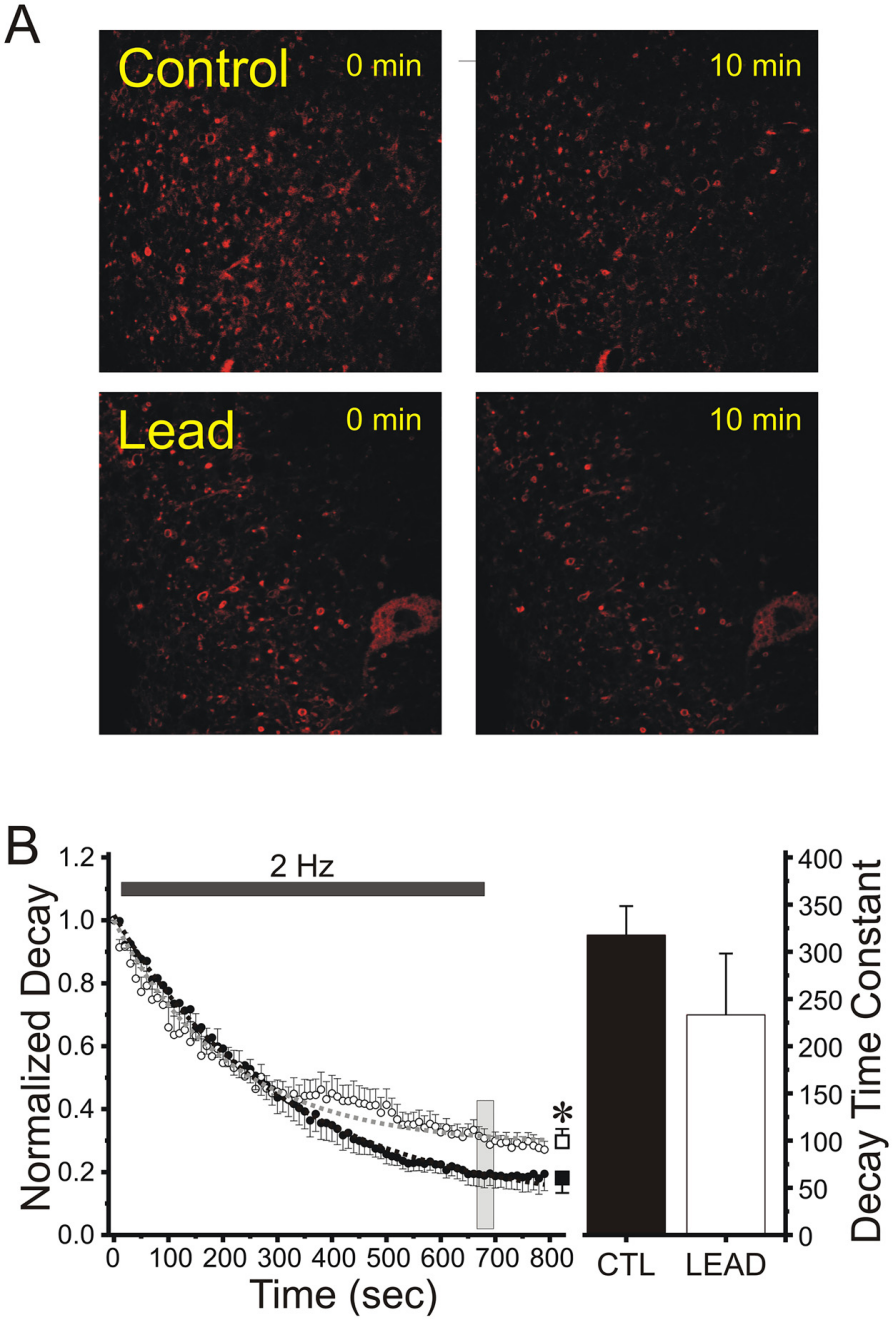
Two-photon laser scanning microscopic (TPLSM) images of FM1-43 vesicular release from Mossy fiber terminals in field CA3 of hippocampal slices confirm that release probability is reduced by chronic Pb^2+^ exposure. (A) Representative TPLSM pseudocolor images of FM1-43 loaded presynaptic terminals in proximal *stratum radiatum* of field CA3c in hippocampal slices from Pb^**2+**^-treated (Lead) and Control rats imaged prior to (0 min) and after 10 minutes (10 min) of 2Hz Mossy fiber stimulation (Calibration Bar: 5 μm). (B) Time course (Mean ± SEM) of stimulus-evoked FM1-43 destaining from large Mossy fiber puncta in field CA3 of hippocampal slices in response to 2Hz Schaffer collateral stimulation (solid bar) in control (solid circles, N = 5 slices, 25 puncta) versus chronically Pb^**2+**^-treated (open circles, N = 6 slices, 27 puncta) rats. Overall, decay time constants were not statistically significantly smaller in slices from Pb^**2+**^-treated rats (white bars) compared to those from control rats (black bars). However, a significant effect of Pb^**2+**^ was observed at the later time points in the 2Hz train (gray bar; * *P* < 0.05, Student’s t-test comparing open and black squares).

While Schaffer collateral-CA1 synapses appear to exhibit a mixture of presynaptic and postsynaptic alterations in expressing long-term synaptic plasticity, mossy fiber-CA3 synapses have been suggested to express largely presynaptic long-term plasticity [35]. We used the same experimental protocol to study vesicular release rates from mossy fiber terminals in field CA3 to determine if the effect of Pb^2+^ exposure on vesicular release was site specific. Fig 3B shows that, while the initial rate of FM1-43 release from mossy fiber terminals was not altered by chronic Pb^2+^ exposure, mean destaining (grey bar indicates values averaged) at the end of the 2Hz stimulus train was reduced (Student’s t-test, *P*<0.05). This reduced destaining late in the stimulus train may suggest reduced rates of vesicle recycling in mossy fiber terminals. Comparison of vesicular release of Schaffer collateral to CA1 synapses versus mossy fiber synapses suggests that Schaffer collateral terminals are more susceptible to effects of early developmental exposure to Pb^2+^ than mossy fiber boutons. Further, the results also suggest that the molecular mechanisms by which Pb^2+^ impairs vesicular release in Schaffer collateral-CA1 synapses are different than those in mossy fiber—CA3 synapses.

### Variance-mean analysis confirms that chronic lead exposure persistently reduces presynaptic release probability at Schaffer collateral terminals

Variance-mean (VM) analysis according to a binomial model of synaptic transmission is a method that has been employed to study a variety of synapses [36,37]. It is mainly applied to steady-state sequences of evoked EPSCs recorded under a variety of conditions by varying extracellular [Ca^2+^], or delivering long repetitive trains of stimulation of different frequencies, each resulting in a range of mean response size with variance that is a parabolic function of *Pr* [38,39,40,41]. In this method, low extracellular [Ca^2+^] yields low *Pr*, release failures and low EPSC variance, high extracellular [Ca^2+^] yields high *Pr*, few failures and, again, low EPSC variance, and physiological extracellular [Ca^2+^] yields intermediate *Pr* and higher EPSC variance. We have recently applied this method to directly estimate presynaptic *Pr* at Schaffer collateral-CA1 synapses during the induction of various forms of LTP and LTD of synaptic transmission [22].

We used three ratios of [Ca^2+^]/[Mg^2+^] in ACSF (4/1, 2/2, and 1/4 mM) to alter *Pr* at Schaffer collateral synapses. Experiments began with establishing stable whole-cell recording from a CA1 pyramidal neuron, and then perfusing the slice with 4/1 [Ca^2+^]/[Mg^2+^] ACSF. Cells were voltage-clamped at -65 mV, and 100 μs constant-current stimulus pulses were delivered to Schaffer collateral/commissural fiber axons every 10 sec to evoke an EPSC. Stable recordings for 8–10 min were made in 4/1 [Ca^2+^]/[Mg^2+^], before replacing the perfusate with 1/4 mM [Ca^2+^]/[Mg^2+^] ACSF. After EPSCs decreased in amplitude and restabilized, which usually took 5–8 min, EPSCs were recorded for an additional 8 min. Slices were then perfused with 2/2 mM [Ca^2+^]/[Mg^2+^] ACSF. After EPSC amplitudes had again stabilized, another 8 min of recordings were made. To ensure that postsynaptic AMPA receptors were responding to a non-saturating concentration of glutamate, as required for VM analysis, experiments were conducted in a low concentration of the selective AMPA receptor antagonist CNQX (100 nM).

**Fig 4:**
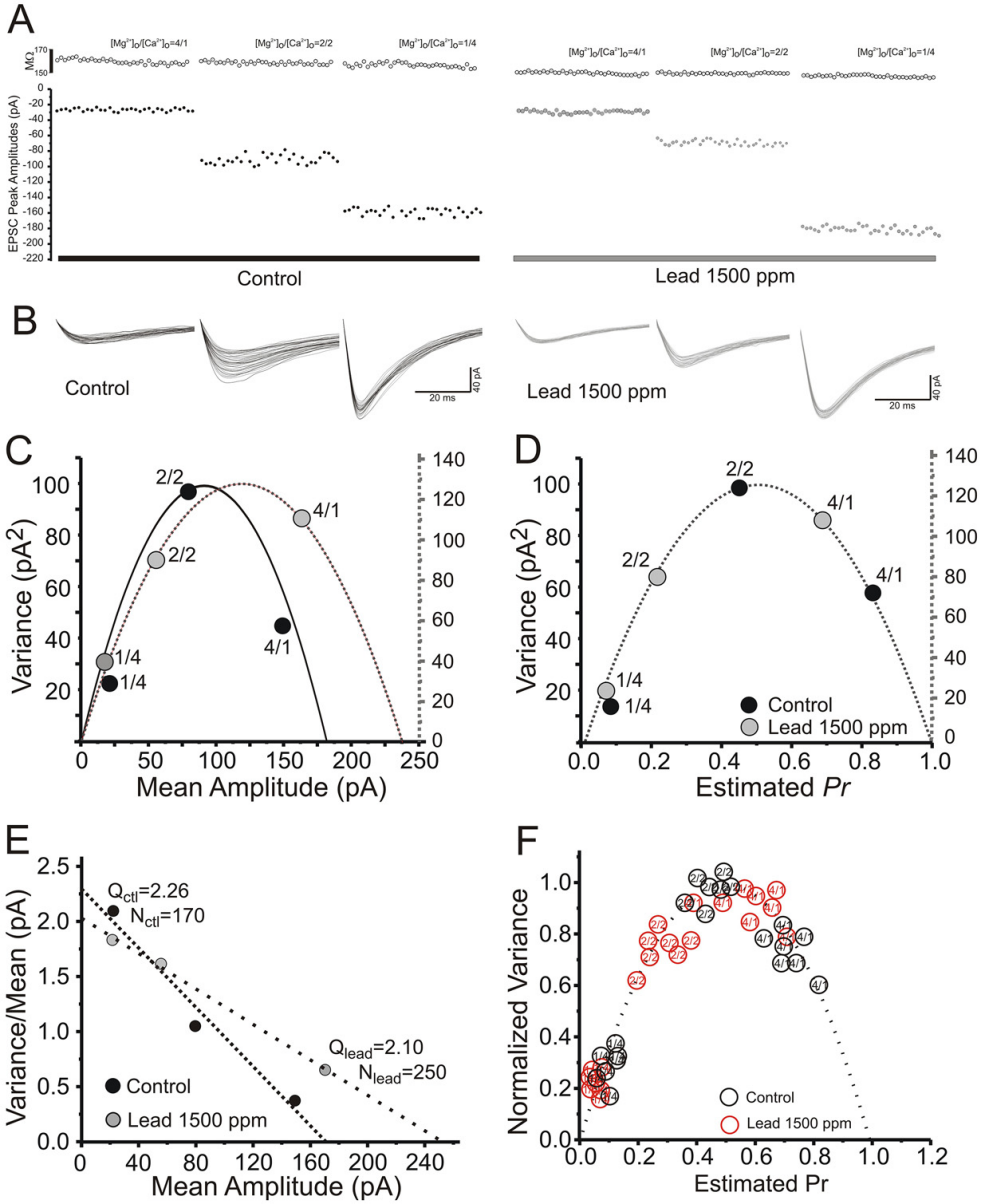
Chronic Pb^2+^ exposure is associated with reduced presynaptic vesicular release probability at Schaffer collateral-CA1 terminals assessed by variance-mean analysis. (A) CA1 pyramidal neuron input resistance (top, in Meg Ω) and Schaffer collateral-evoked EPSC peak amplitudes (bottom, in pA) recorded at 1, 2 and 4 mM [Ca^**2+**^]_o_, in hippocampal slices from a control (left) and chronic Pb^**2+**^-exposed (right) slice. (B) EPSC trace envelopes in CA1 pyramidal neurons in a slice from a Control (left) and a chronic Pb^**2+**^-treated (right) rat. (C) Variance-mean relationships determined by altering [Ca^**2+**^]_o_ to 1, 2 and 4 mM in CA1 pyramidal neurons in slices from control (black circles; N = 8) and Pb^**2+**^-treated (grey circles; N = 7) rats. (D) Data from (C) corrected to estimate *P*_*r*_, showing mean ± SEM of mean-variance points in slices from control (black circles) and Pb^**2+**^-treated (grey circles) rats normalized to the maximal peak amplitude recorded at 4 mM [Ca^**2+**^]_o_. Data from control and Pb^**2+**^-exposed slices were both well fit by a single parabola forced to pass through 0,0 with grey circles shifted to the left, consistent with a presynaptic reduction in *P*_*r*_ from chronic Pb^**2+**^ exposure. (E) Plot of variance/mean ratio versus mean EPSC amplitude (pA) from a single representative slice, which converts the parabolic relationship between mean and variance to a linear one. The number of release sites (N) was derived by estimating the slope of the linear fit, while the y-intercept denotes the quantal size (Q) of the EPSC. The reduction in slope indicates that chronic Pb^**2+**^ exposure was associated with a reduction in presynaptic *P*_*r*_. (F) Individual variance-mean data points for each control slice (black circles) and each Pb^**2+**^-exposed slice (red circles) at each [Ca^**2+**^]_o_.

Fig 4 illustrates experiments using VM analysis to determine the long-term effects of Pb^2+^ exposure on *Pr*. Fig 4A shows individual CA1 pyramidal neuron membrane resistance and EPSC amplitudes recorded in representative slices from a control and a Pb^2+^-exposed rat, during the stable periods after each change of extracellular [Ca^2+^]_o_. The stability of the data recorded was assessed by fitting a straight line to the amplitudes in each recording condition and plotted versus repetition number. For analysis, only data that displayed less than 20% change in the regression line over at least 30 data points were selected for further VM analysis [39]. Fig 4B shows representative envelopes of individual EPSCs at 1/4mM (left), 2/2mM (center) and 4/1mM (right) [Ca^2+^]/[Mg^2+^] ratios in a slice from a control and a Pb^2+^-exposed rat. As shown in panel 4C, the VM relationship obtained by varying extracellular [Ca^2+^] is parabolic, with maximum variance at the peak of the parabola. In pyramidal hippocampal neurons from Pb^2+^-exposed rats, individual slice data point (Fig 4F) and mean amplitudes (Fig 4D) at different [Ca^2+^], converted to *Pr*, were reduced along the same parabolic fit at all three [Ca^2+^]_o_. (*P*<.0.01, Student’s t-test), consistent with a reduction in presynaptic release probability compared to controls. Panel 4E shows a typical associated variance/mean versus mean linear plot, where the linear fits of pyramidal neurons from control and Pb^2+^-exposed rats significantly differed in slope (*P*<0.05, Student’s t-test), consistent with a presynaptic site of reduced *Pr*. Across all experiments (N = 7 for each treatment group), *Pr* calculated by this method was significantly reduced in slices from Pb^2+^-exposed rats at low (1/4 mM; Pb^2+^ = 0.037 ± 0.0013, control = 0.051 ± 0.0041, *P*<0.05, Student’s t-test), medium (2/2 mM; Pb^2+^ = 0.28 ± 0.028, control = 0.45 ± 0.02, *P*<0.05, Student’s t-test) and high (4/1 mM; Pb^2+^ = 0.60 ± 0.026, control = 0.72 ± 0.023, *P*<0.05, Student’s t-test) [Ca^2+^]/[Mg^2+^] ratios. These reductions in *Pr* were not associated with significant changes in number of release sites (N) or quantal size (Q).

### Chronic lead exposure does not produce marked changes in presynaptic calcium influx into Schaffer collateral terminals

**Fig 5:**
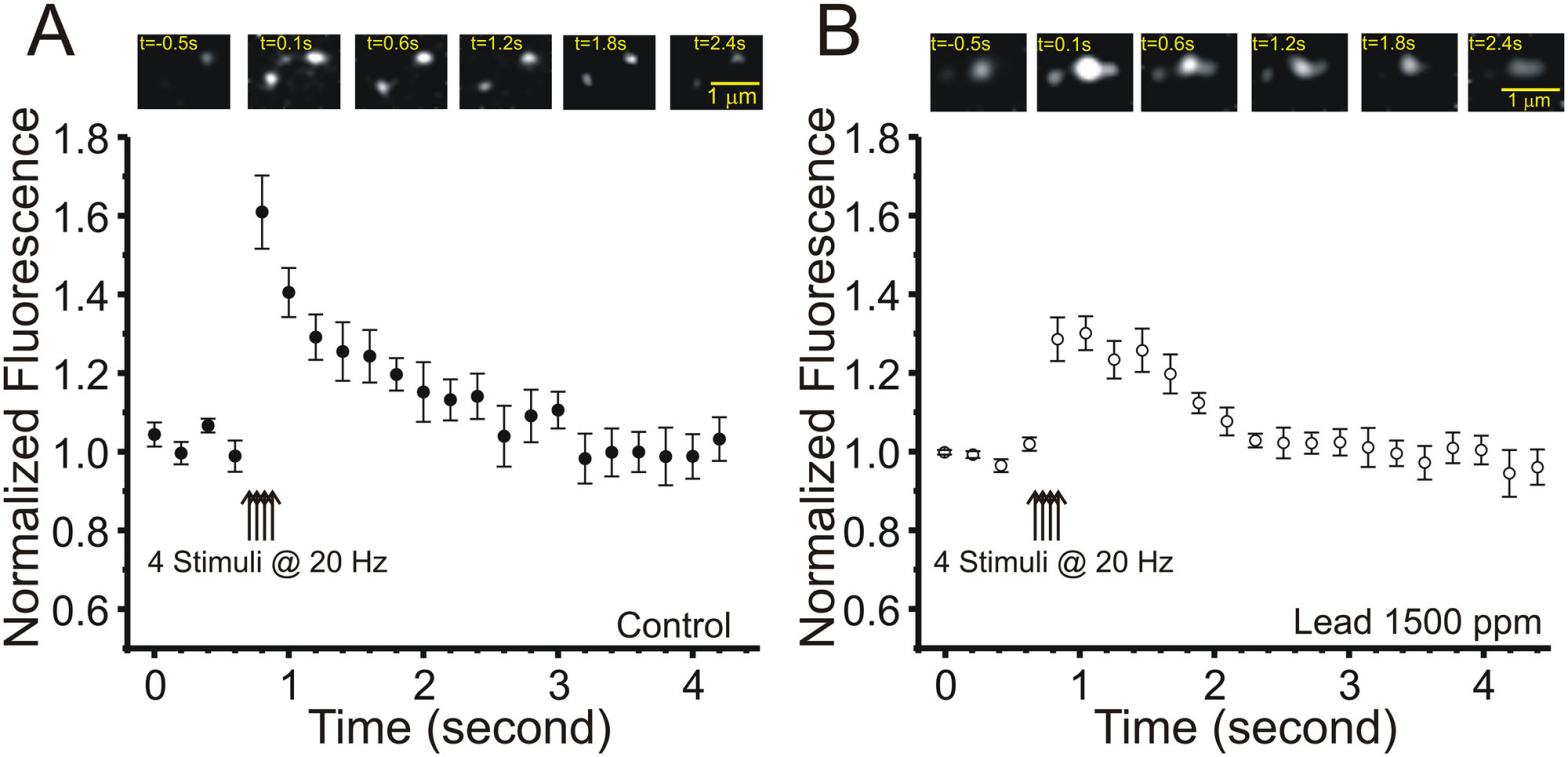
Chronic Pb^2+^ treatment does not produce marked changes in presynaptic Ca^2+^ influx into Schaffer collateral terminals. Mean ± SEM presynaptic stimulus-evoked Mg^**2+**^-Green fluorescence increases in presynaptic terminals in response to a burst of Schaffer collateral stimuli (4x20Hz) in slices from control (A, filled circles, N = 10 control slices, 25 terminals) versus Pb^**2+**^-exposed (B, open circles, N = 13 slices, 32 terminals) rats (insets above are single fluorescent spines from a control and Pb^**2+**^-exposed slice at the indicated time points post-stimulation).

Calcium channels (P/Q and N-type) are the major source of action potential mediated Ca^2+^ influx into presynaptic terminals and previous studies have shown that Pb^2+^ inhibits calcium channels in cultured cells, an effect that is reversible by washing the cellular preparation [42]. Therefore, if Pb^2+^ exposure chronically alters the activity of these channels, this could indirectly alter *Pr* of synaptic vesicles. To directly test whether chronic Pb^2+^ exposure produces a persistent inhibition of presynaptic Ca^2+^ influx, we injected Mg^2+^ Green-AM, a calcium indicator dye that is membrane-permeable [43], directly into stratum radiatum of field CA1 of hippocampal slices. Mg^2+^ Green positive fluorescent puncta were visualized in field CA1 using two-photon excitation (Fig 5 insets). Fig 5 demonstrates the kinetics of Mg^2+^ Green fluorescence increases in response to a 100Hz burst of four Schaffer collateral stimuli. We have shown previously that these responses persist in the presence of NMDA and AMPA receptor antagonists, despite the loss of fEPSPs, are blocked by cadmium and omega conotoxin, and co-localize with FM4-64, confirming a presynaptic nature for these calcium transients [44].

Comparison of the mean fluorescent time courses of stimulus-evoked presynaptic Ca^2+^ influx transients in Schaffer collateral terminals in slices from control (Fig 5A) versus Pb^2+^-exposed (Fig 5B) rats revealed that these transients were almost completely superimposable, with the exception of the first two peak fluorescence time points, which were significantly larger in amplitude in control slices in the first 400 milliseconds (repeated measures ANOVA, F(1,20) = 12.747; p = 0.0019), but not different for the rest of the transients (repeated-measures ANOVA F(1,20) = 0.097; p = 0.7588). Overall, the area under the curves for these two sets of terminals were not significantly different (*P* > 0.05), suggesting that reductions in presynaptic [Ca^2+^] influx are not likely to be responsible for the marked reduction in vesicular release associated with chronic Pb^2+^ exposure. Taken together, our data indicate that chronic exposure to Pb^2+^ during early development results in a persistent reduction in presynaptic *Pr* that is likely due to actions downstream of presynaptic Ca^2+^ influx at the level of SNARE protein-mediated exocytosis.

### Chronic in vivo exposure to lead resulted in a reduction in number of ready releasable pool/docked vesicles and a dispersion of vesicles in the resting pool in Shaffer Collateral terminals measured by transmission electron microscopy

**Fig 6:**
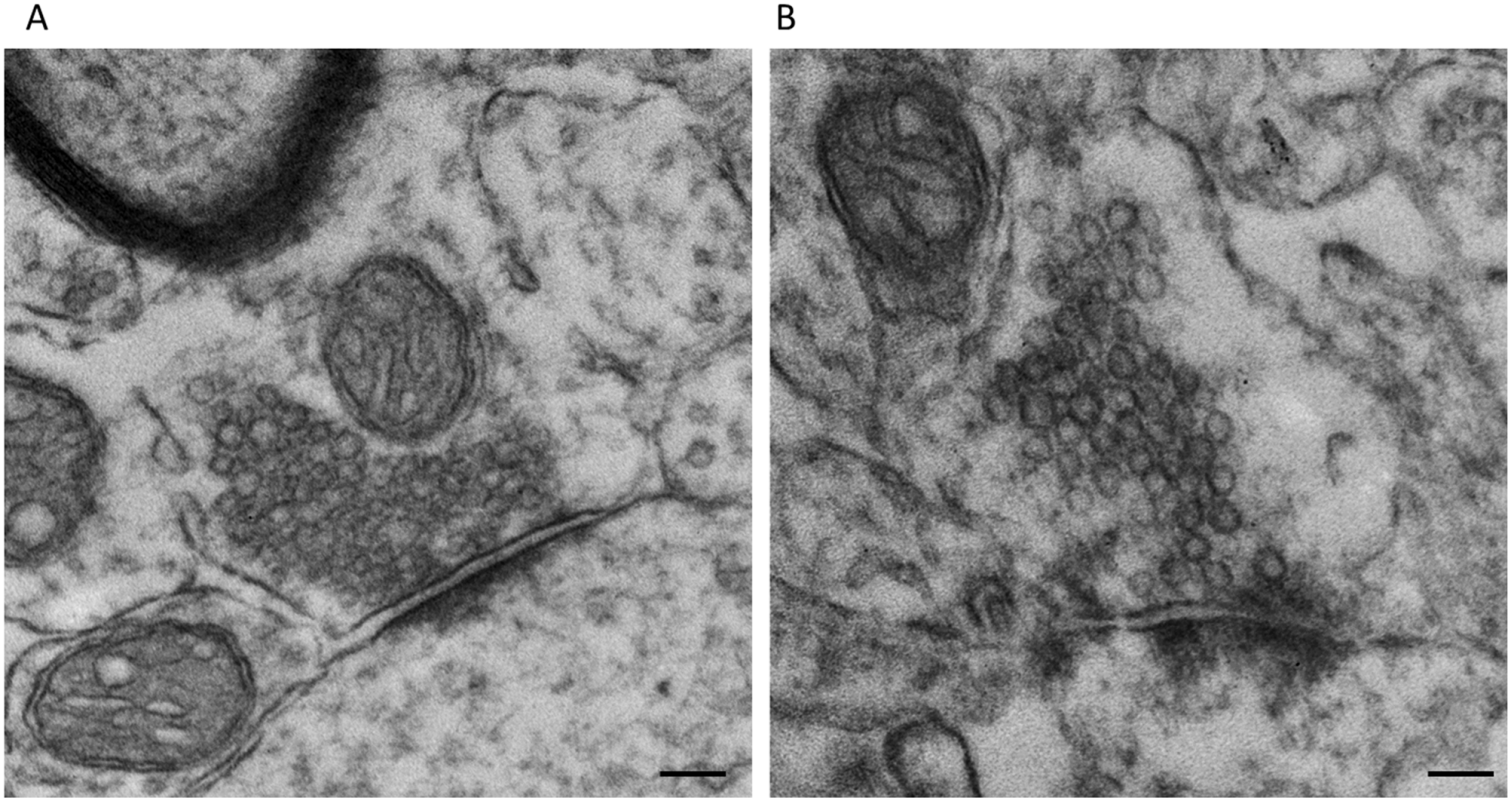
Transmission electron micrographs of Schaffer Collateral terminals. Simple, asymmetric, Collateral—CA1 synapses from control (A) and Pb^**2+**^ exposed (B) animals were imaged and examined using transmission electron microscopy. Scale bar = 100 nm.

**Fig 7:**
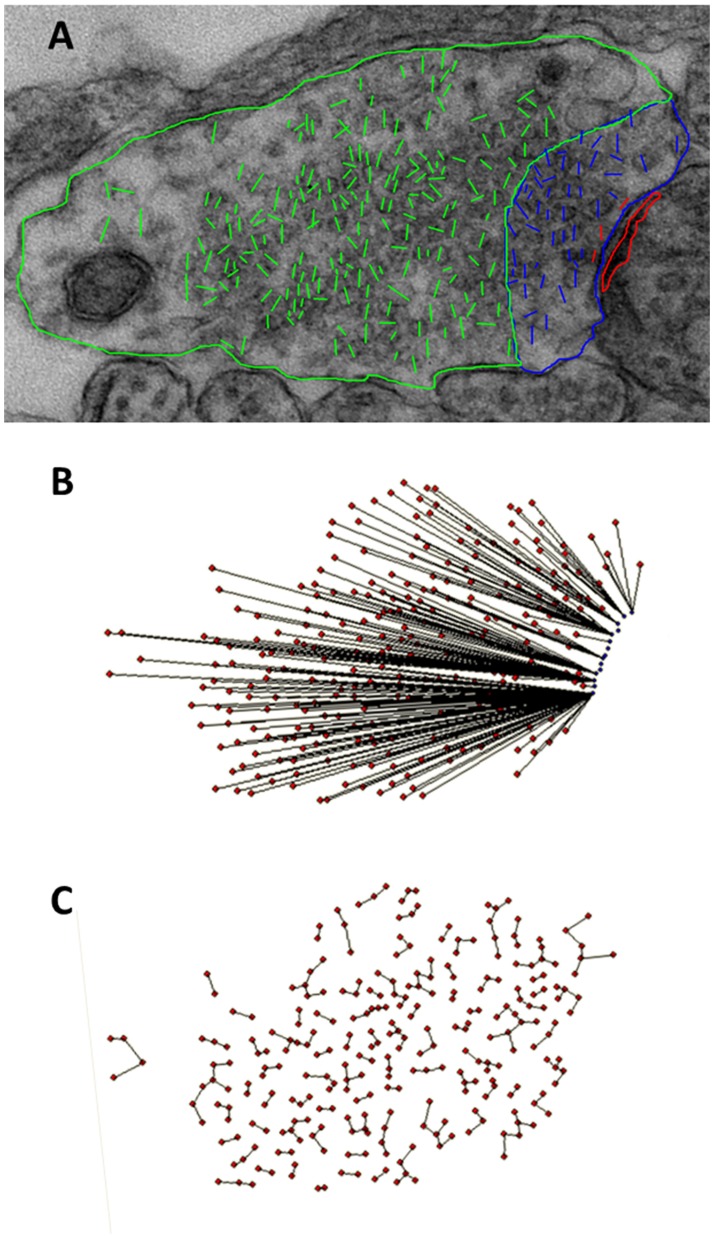
ImageTool and LoClust were used in the measurement of vesicles in pre-synaptic terminals. (A) An axon terminal with colors to indicate quantification of vesicles and measurements. Docked vesicles contact the pre-synaptic active zone (PAZ). Their diameter is marked in red. Rapidly recycling vesicles are those that are found within 200 nm from the PAZ. Their diameter is marked in blue. Resting vesicles are found more than 200 nm away from the PAZ. Their diameter is marked in green. The length of line represents the diameter of the vesicle. (B) LoClust measurements of vesicle distance to the PAZ. The center of each vesicle to the closest PAZ point was determined using LoClust. (C) The nearest-neighbor distance was the distance found between the center points of the nearest two vesicles, as determined by LoClust.

Previous studies from our laboratory using hippocampal neuronal cultures have shown that the effect of Pb^2+^ on vesicular release was specific to a fast-releasing pool of vesicles that we hypothesized to be associated with the readily-releasable vesicle pool [19]. Therefore, to directly confirm that Pb^2+^ inhibition of vesicular release may be mediated by alterations in the readily-releasable/docked vesicle pool, we used TEM to visualize presynaptic axon terminals of control versus Pb^2+^-exposed rats to analyze vesicle position (Fig 6). We classified vesicles as: 1) part of the readily-releasable/docked vesicle pool if they were physically contacting the presynaptic active zone (PAZ), 2) part of the rapidly recycling pool if their center was within 200 nm of the PAZ, or 3) part of the reserve pool if their vesicular center was greater than 200 nm from the PAZ (Fig 7) based on previous demonstration of three pools of vesicles [11,12]. Our results indicate marked changes in the vesicular pools of presynaptic terminals in the CA1 region of the hippocampus from rats that were exposed to Pb^2+^ relative to controls (Table 1). That is, Pb^2+^ exposure resulted in a significant reduction in the number of readily-releasable/docked vesicles in Shaffer Collateral terminals (p = 0.034, Table 1, IA).

**Table 1:** Summary of Schaffer Collateral-CA1 Transmission Electron Microscopy (TEM) Results.

	Control(Mean ± SEM)	Pb^2+^(Mean ± SEM)	P value
**I. Raw Vesicle Counts**			
A) Rapidly Releasable/Docked Vesicle Pool	7.350 ± 0.5333	4.635 ± 2.426	0.0348
B) Recycling Vesicle Pool	22.20 ± 3.575	22.19 ± 4.936	0.4496
C) Resting Vesicle Pool	44.67 ± 6.881	60.55 ± 6.192	0.2382
D) Total Number of Vesicles	118 ± 31.15	147 ± 38.42	0.2927
**II. Vesicular Diameters**			
A) Rapidly Releasable/Docked Diameter	20.09 ± 1.211	20.23 ± 1.312	0.4689
B) Recycling Vesicle Diameter	23.79 ± 1.116	25.33 ± 0.8911	0.3133
C) Resting Vesicle Diameter	25.76 ± 0.5456	24.68 ± 1.270	0.2289
D) Average Diameter of all vesicles	23.88 ± 0.4596	24.20 ± 0.4270	0.0623
**III. Length Measurements**			
A) PSD length	317.7 ± 18.08	297.3 ± 5.4	0.1655
B) PAZ length	309.7 ± 13.58	296.2 ± 14.93	0.5214
**IV. Nearest Neighbor Distance (Clustering)**			
A) Rapidly Releasable/Docked + Recycling (0–200 nm)	41.28 ± 0.8975	42.61 ± 0.9356	0.3125
B) Resting (201–500 nm)	43.75 ± 0.7838	46.49 ± 0.6697	0.0017
**V. Mitochondrial Measurements**			
A) Terminals with mitochondria	15.80 ± 3.153	14.00 ± 2.121	0.3251
B) Terminals with multiple mitochondria	4.200 ± 0.9695	2.600 ± 0.9695	0.0001
C) Total number of mitochondria	23 ± 7.095	21.00 ± 8.660	0.4348
D) Diameter of mitochondria	208.4 ± 5.535	215.7 ± 14.66	0.4643
E) Large mitochondria (300 nm or greater)	0.3333 ±0.3333	2.667 ± 0.8819	0.0659

Mean, SEM and p values for each TEM analysis are summarized in this table. All analyses were done using a T-test with Welch Correction for unequal variances.

We also measured vesicle nearest-neighbor distance as a function of distance from the PAZ (Fig 7B). The distance between the centers of each vesicle was marked and the nearest neighbor distance between vesicles measured (Fig 7C). The distance from the PAZ to each vesicle was binned and arranged into two clusters representing (1) readily-releasable/docked plus recycling pools of vesicles (0–200 nm from PAZ) and (2) the reserve vesicle pool (201–500 nm from PAZ). Schaffer Collateral terminals of Pb^2+^-exposed animals did not show significant changes in nearest-neighbor distance between vesicles of the RRP/docked pools plus recycling pools, but did show a highly significant increase in nearest neighbor distance between vesicles of the reserve pool (Table 1, IVB; p = 0.0017). This data suggests there are alterations in clustering of vesicles in the reserve pool that may alter vesicle movement and availability, contributing to the significant reduction in functional release probability and synaptic transmission produced by chronic Pb^2+^ exposure.

### Effect of chronic lead exposure on presynaptic mitochondria number and size

To determine if changes in vesicular release and vesicle clustering were related to energy availability in axon terminals, we examined the number of mitochondria in presynaptic terminals from the TEM images. Exposure to Pb^2+^ had no significant effect in the number of terminals containing at least one mitochondrion (Table 1, VA) but there was a marked decrease in the number of terminals that contained multiple (>2) mitochondria in the Pb^2+^-exposed group relative to controls (Table 1, VB) suggesting that trafficking of mitochondria to presynaptic terminals may be altered by chronic Pb^2+^ exposure. There were no significant differences in total mitochondria counted (Table 1, VC), and the mean diameter of presynaptic terminal mitochondria was similar between control and Pb^2+^-exposed groups (Table 1, VD), although there was a nearly significant increase in the number of mitochondria with diameter greater than 300 nm (Table 1, VD). These findings suggest that mitochondrial function and energy availability in the form of ATP in presynaptic terminals may be decreased by Pb^2+^ exposure.

## Discussion

Our present findings in hippocampal brain slices containing relatively intact local synaptic networks, supply multiple lines of evidence that chronic *in vivo* exposure to Pb^2+^ during development results in reductions in presynaptic vesicular release, an effect that is associated with ultrastructural changes in the presynaptic terminals of young adult rats. At Schaffer collateral-CA1 synapses in the hippocampus, paired-pulse facilitation, VM analysis and direct multi-photon imaging of vesicular release using FM1-43 all indicate a persistent reduction in presynaptic vesicular release probability in Pb^2+^-exposed young adult rats. These findings are consistent with our previous *in vitro* studies demonstrating a marked impairment in presynaptic vesicular release using FM1-43 by Pb^2+^ in hippocampal neuron cultures [19]. TEM analysis revealed that these functional impairments in vesicular release were associated with fewer vesicles in the readily-releasable/docked vesicle pool (Table 1, IA). In the CA1 region, presynaptic terminals from Pb^2+^-exposed rats had a significantly lower number of terminals that contained two or more mitochondria suggesting that the amount of ATP available for release mechanisms and vesicle movement may be significantly reduced by Pb^2+^ exposure.

Synaptic mitochondria play an important role in synaptic transmission, organization and movement of vesicles in the reserve pool to the readily releasable pool, and calcium buffering and homeostasis [42,14]. Therefore, it is likely that the impairment in vesicular release in Pb^2+^-exposed animals that we have identified in the present study may be due, at least in part, to reduced ATP availability resulting from a reduced number of presynaptic terminal mitochondria. Our TEM studies also showed that Pb^2+^ exposure altered the clustering of vesicles in the resting pool by increasing vesicle nearest-neighbor distance (Fig 7). Longer distance between vesicles in the reserve pool may be associated with reduced vesicular release. In mice deficient in the neural adhesion molecule L1, there was a marked increase in the nearest-neighbor distance between vesicles. These mice had a higher number of failures in transmitter release [45]. Other TEM studies have shown that synaptic vesicle clustering occurs via connectors of different sizes, reflecting a diffuse intervesicular matrix [46,47]. Furthermore, the brain-specific phosphoprotein synapsin plays an important role in clustering and movement of vesicles in the reserve pool in a phosphorylation-dependent manner [47,48,49]. We have previously shown that synapsin I phosphorylation at sites 4 (serine 62) and 5 (serine 67) were significantly decreased by Pb^2+^ exposure with no effect on total synapsin I protein levels [21]. Thus, it is possible that the increase in vesicle nearest-neighbor distance that we have found in the reserve vesicle pool of presynaptic CA1 terminals from Pb^2+^-exposed animals may be the result of lower levels of synapsin I phosphorylation leading to alteration in synapsin dimerization, increasing vesicle interconnector length and thus, increasing vesicle nearest-neighbor distance in the resting pool. Consistent with this idea, synapsin I deficient mice have been shown to exhibit dispersed vesicles [50], and decreased phosphorylation of synapsin I by cdk5 at site 7 (serine 551) has been shown to disrupt synaptic vesicle clustering and increase vesicle nearest-neighbor distance [51]. Therefore, although we have not assessed the effect of Pb^2+^ exposure on synapsin-serine 551 phosphorylation, it is clear that phosphorylation at this site is both necessary and sufficient to alter vesicle clustering in the reserve pool and increase nearest-neighbor distance.

What are potential mechanisms of Pb^2+^-induced impairments in vesicular release? Previous studies using acute exposure to Pb^2+^ in cultured cells have shown inhibition of Ca^2+^ channels by Pb^2+^, an effect that is reversible by washing the cellular preparation [41]. On the other hand, there could be additional mechanisms by which Pb^2+^ may alter vesicular release by changes at the level of SNARE proteins as we have previously shown [19,20]. Therefore, we needed to assess whether the impairment of vesicular release that we have documented here in chronically Pb^2+^-exposed animals could be directly due to chronic inhibition of Ca^2+^ channel function that persists upon Pb^2+^ washout, or whether chronic Pb^2+^-exposure results in additional changes downstream of Ca^2+^ influx that persist in the absence of extracellular Pb^2+^. To directly measure the effect of *in* vivo Pb^2+^ exposure on presynaptic calcium entry, we directly measured Ca^2+^ influx into Schaffer collateral-CA1 terminals. Fluorescent imaging of presynaptic Ca^2+^ influx showed little change in amplitude or duration of voltage-dependent Ca^2+^ channel-mediated Ca^2+^ entry, though the reduction in the first two time points of the transients suggests that channel kinetics may be somewhat altered. Thus, our slice experiments support the presence of additional effects of chronic developmental exposure to Pb^2+^ that are downstream of Ca^2+^ entry, somewhere at the level of vesicular SNARE protein-mediated docking, recycling, or even affecting the long-term stability of the release complex, as we have previously shown in hippocampal neuron cultures [19].

The correlation between functional and ultrastructural alterations in Pb^2+^-exposed animals was striking, and is strongly suggestive of profound alterations in SNARE protein levels and function that resulted in both a functional impairment in the vesicular release process, and a reduction in the number of readily-releasable/dock vesicles. While these changes in excitatory glutamatergic Schaffer collateral terminals are clear, it is by no means assured that the same pattern of alterations occurs at all presynaptic terminals in the brain. Indeed, a pressing question is whether inhibitory GABAergic terminals are affected similarly in magnitude and direction by chronic developmental exposure to Pb^2+^, since such alterations could have important effects on cognition and propensity for seizures. To this aim we should note a recent study using stereological cell counting data indicated that the number of parvalbumin-positive GABAergic interneurons was significantly reduced in number in the hippocampus of rats of similar age and Pb^2+^ exposure as our present study [52].

In this study, rats were exposed to Pb^2+^ chronically during gestation, postnatally and continuing through to young adulthood. The preparation of brain slices for the studies did not contain Pb^2+^ in the artificial cerebrospinal fluid used to maintain slice viability during experiments, suggesting that the effects observed were of a developmental nature. This leads to the question of whether removal of Pb^2+^ *in vivo* could allow recovery of normal presynaptic function, and whether there are treatments that might protect against these effects of Pb^2+^ exposure. Neal et al. [19] have shown that BDNF synthesis and release are decreased by exposure of hippocampal neurons in culture to Pb^2+^, an effect associated with reductions in the levels of SNARE proteins and inhibition of vesicular release. These effects of chronic Pb^2+^ exposure were rescued by the exogenous addition of BDNF. Moreover, Stansfield et al. [21] using the same Pb^2+^ exposure paradigm of hippocampal neurons in culture showed that Pb^2+^ also impairs the transport of BDNF-containing vesicles possibly by altering Huntingtin phosphorylation at a site that promotes anterograde BDNF vesicle movement. This effect of Pb^2+^ results in impaired BDNF release, decreasing TrkB activation, and leading to decreased phosphorylation of synapsin I. Together, these studies strongly suggest that BDNF and treatments such as enriched environments that increase BDNF levels and release, may rescue the effects of Pb^2+^ that we observed *in vivo* in intact hippocampal slice synaptic networks. In fact, previous studies from our laboratory have shown that environmental enrichment is able to reverse the Pb^2+^-induced impairment of spatial learning deficits in rats of similar age and Pb^2+^ treatment [53]. Ongoing studies will investigate whether enhancing BDNF-TrkB signaling by pharmacological approaches or using environmental enrichment paradigms can reverse the Pb^2+^-induced impairment of vesicular release and presynaptic structural changes that we have documented in the present study.
